# Supplementary material for: Evidence for Prepulse Inhibition of Visually Evoked Motor Response in Drosophila melanogaster
Source: Biology (Basel). 2023 Apr 21;12(4):635. doi: 10.3390/biology12040635 (PMC10135638; doi:10.3390/biology12040635)
Supplement: Supplementary file 1 [file biology-12-00635-s001.zip › Supplementary Files/Supplementary Figures.docx]

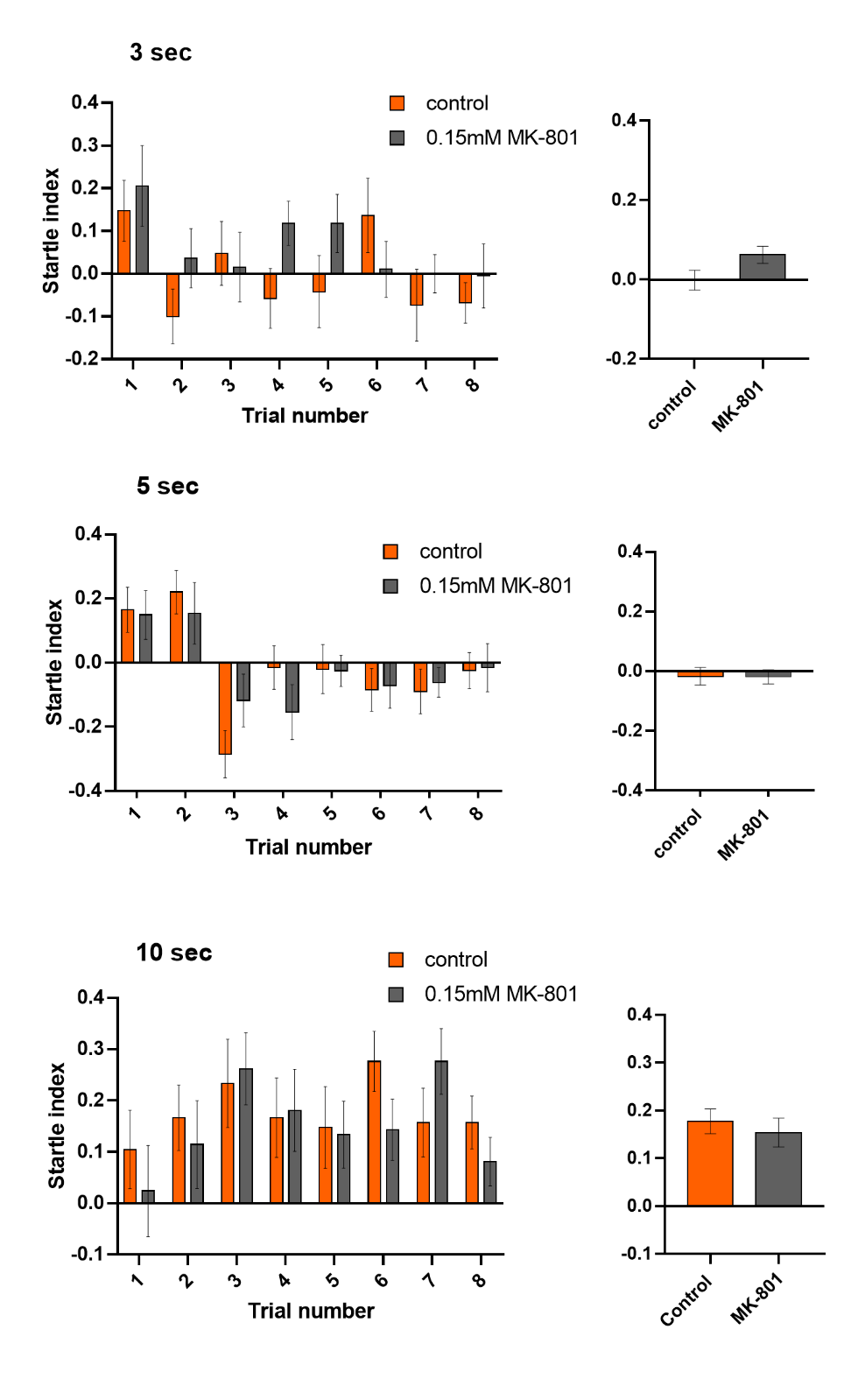


**Supp. Figure 1. Effects of 0.15 mM MK-801 on prepulse inhibition.** Flies treated with 0.15 mM MK-801 or control solution presented to prepulse stimuli before the startle-inducing lights-off stimuli, 3 (upper panel), 5 (middle panel), or 10 (lower panel) seconds apart. Averages of all trials for MK-801 exposure and control groups are shown in the respective right panels. For the prepulse interval of 3 seconds, no significant effects from MK-801 exposure, trials or interactions to the movement response were observed (p_MK-801_=0.056, p_trials_=0.062, p_interaction_=0.450). For a 5-second prepulse interval, only a significant effect from trials was observed (p_MK-801_=0.977, p_trials_=0.0001, p_interaction_=0.634). For the 10-second prepulse interval, again, no effects were observed from the tested variables (p_MK-801_=0.540, p_trials_=0.178, p_interaction_=0.726).


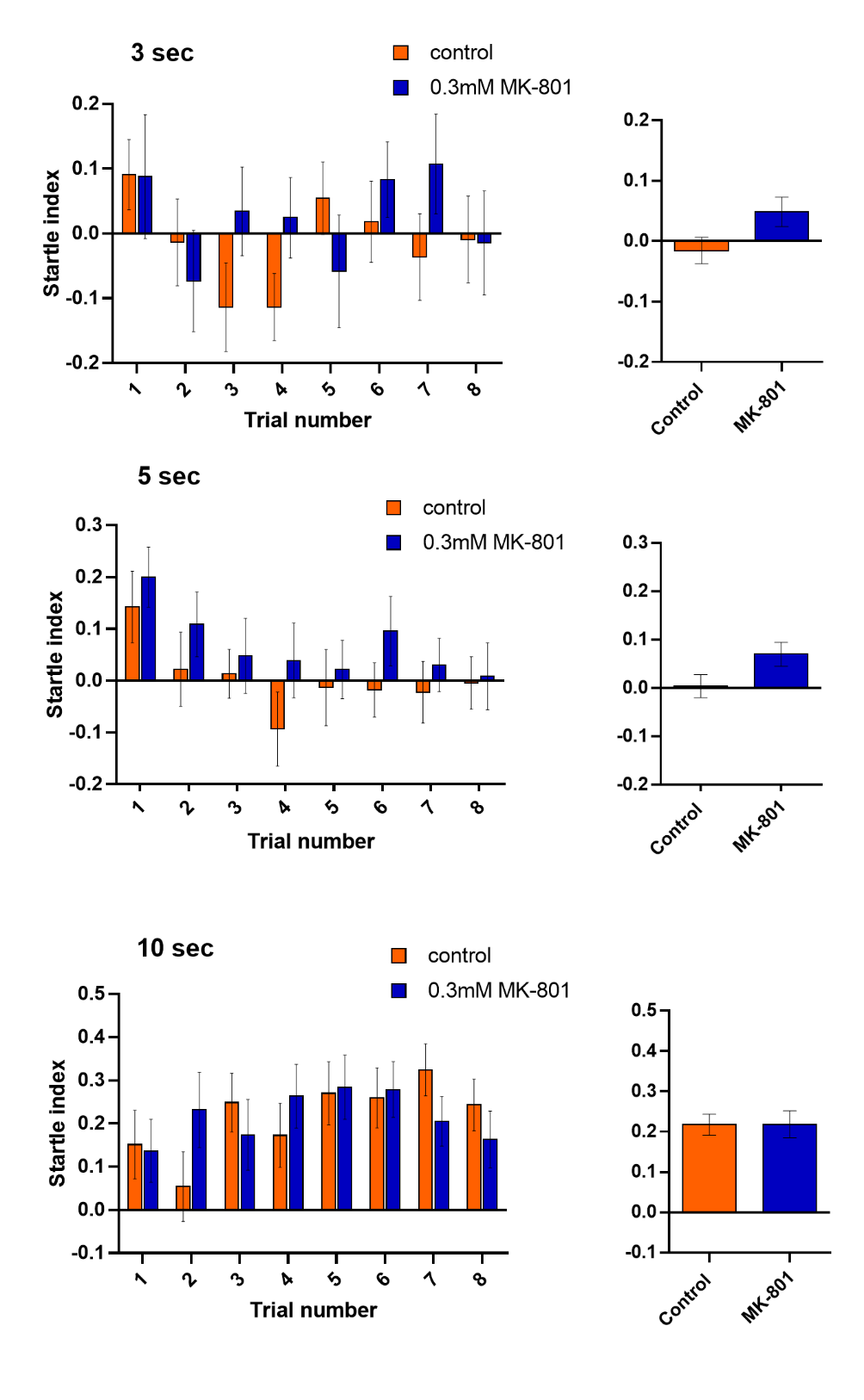


**Supp. Figure 2. Effects of 0.3 mM MK-801 on prepulse inhibition.** Flies treated with 0.3 mM MK-801 or control solution presented to prepulse stimuli before the startle-inducing lights-off stimuli, 3 (upper panel), 5 (middle panel), or 10 (lower panel) seconds apart. Averages of all trials for MK-801 exposure and control groups are shown in the respective right panels. For a 3-second prepulse interval, no significant effects from MK-801 exposure, trials or interactions to the movement response were observed (p_MK-801_=0.054, p_trials_=0.154, p_interaction_=0.426). Likewise, for the 5-second prepulse interval, no variable showed a significant effect (p_MK-801_=0.057, p_trials_=0.062, p_interaction_=0.980). For the 10-second prepulse interval, again, no effects were observed from the tested variables (p_MK-801_=0.971, p_trials_=0.332, p_interaction_=0.452).


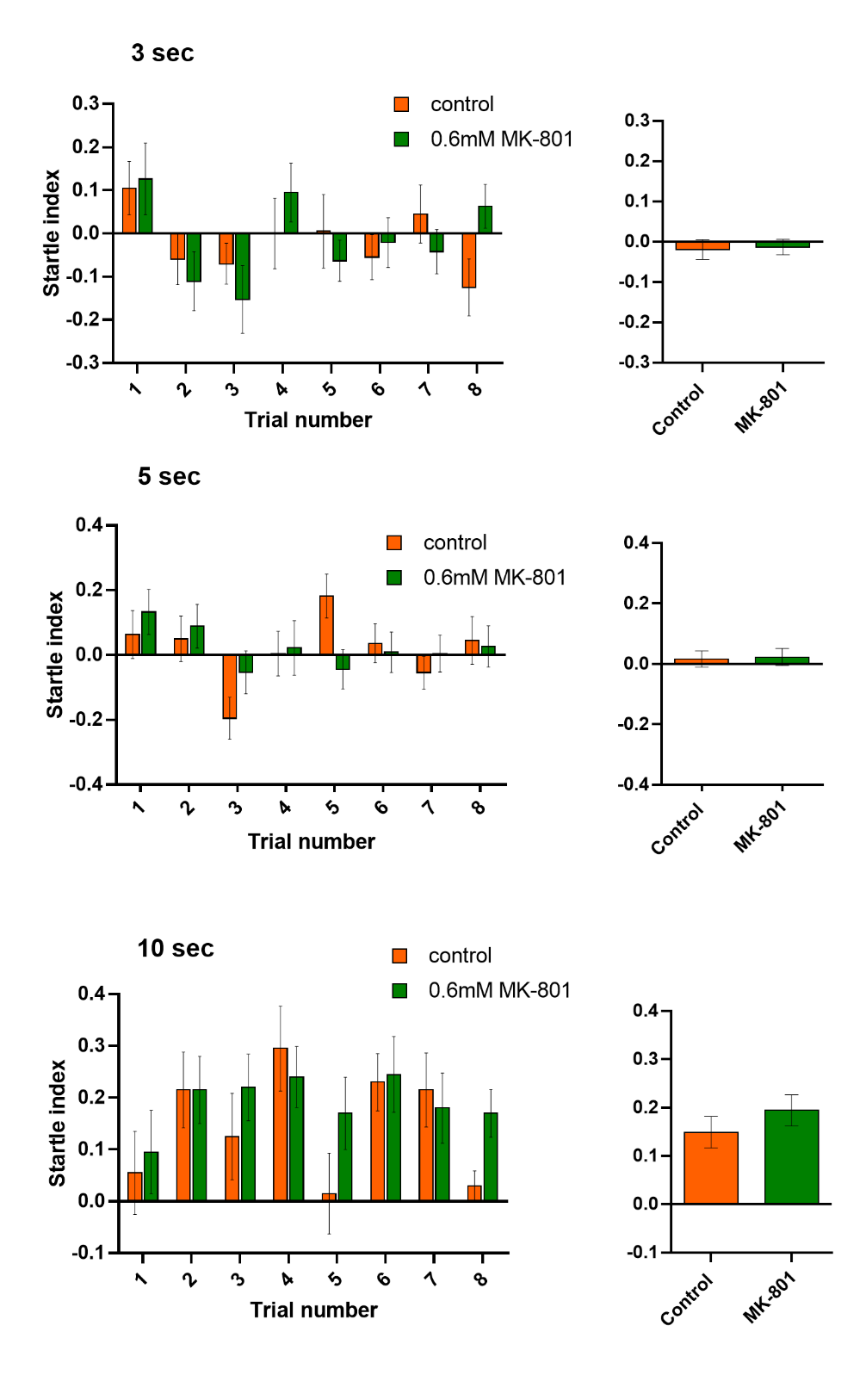


**Supp. Figure 3. Effects of 0.6 mM MK-801 on prepulse inhibition.** Flies treated with 0.6 mM MK-801 or control solution presented to prepulse stimuli before the startle-inducing lights-off stimuli, 3 (upper panel), 5 (middle panel), or 10 (lower panel) seconds apart. Averages of all trials for MK-801 exposure and control groups are shown in the respective right panels. For a 3-seconds prepulse interval, only a significant effect from trials was observed (p_MK-801_=0.842, p_trials_=0.020, p_interaction_=0.355). Likewise, for the 5-second prepulse interval, a significant effect from trials was observed, but not MK-801 exposure, trials or interaction (p_MK-801_=0.862, p_trials_=0.024, p_interaction_=0.204). For the 10-second prepulse interval, the trials were again the only variable significatively influencing movement response (p_MK-801_=0.334, p_trials_=0.016, p_interaction_=0.650).


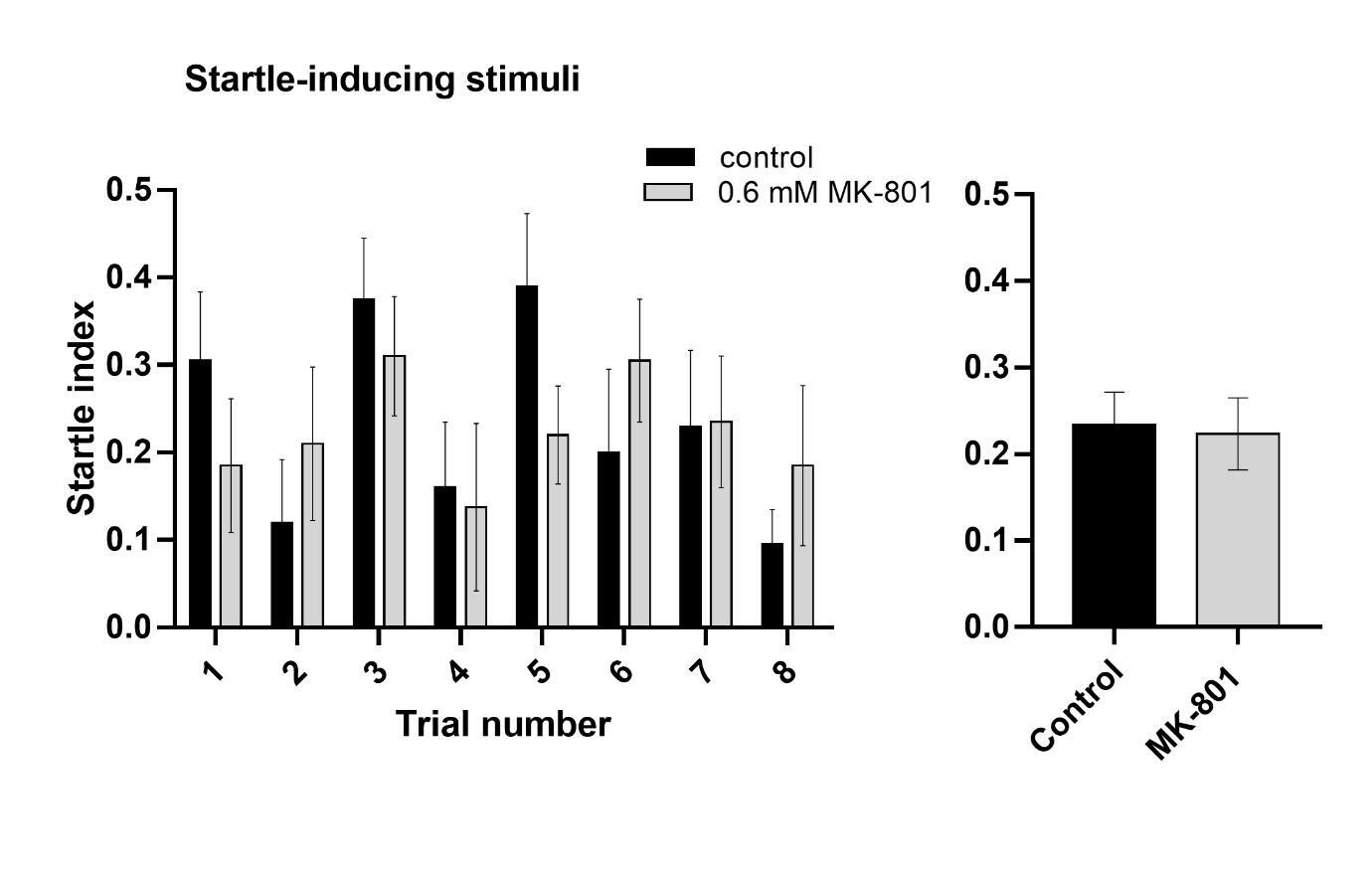


**Supp. Figure 4. Response to lights-off stimuli after MK-801 administration.** The effects of 0.6 mM MK-801 exposure on the startle index were measured following the same protocol shown in Figure 1. No significant differences between treatments were observed, the trial number variable was shown to be significant, but not the treatment-trial interaction (two-way ANOVA, p_MK-801_ =0.846, p_trials_=0.039, p_interaction_=0.399, n= 8/group). Both group averages also showed a significantly increased motor response (one-sample t-test, p<0.0001 for controls and MK-801-exposed flies)
